# Supplementary material for: Complexome profiling on the Chlamydomonas lpa2 mutant reveals insights into PSII biogenesis and new PSII associated proteins
Source: J Exp Bot. 2021 Aug 26;73(1):245–62. doi: 10.1093/jxb/erab390 (PMC8730698; doi:10.1093/jxb/erab390)
Supplement: erab390_suppl_Supplementary_Dataset_S1 [file erab390_suppl_supplementary_dataset_s1.zip › Supplemental Dataset 1 - Excel List and all profiles/plots/PFL1_Cre01.g044800.html]

Trivial name: PFL1  
  
Euclidean distance: 155877.70  
Mean Intensity (WT): 42176.20  
Mean Intensity (Mut): 26065.49  
Distance: 3.70  
  
MapMan:   
  
p value of intensity sums Welch test: 0.4483
